# Promoting Executive Function Skills in Preschoolers Using a Play-Based Program

**DOI:** 10.3389/fpsyg.2021.720225

**Published:** 2021-12-29

**Authors:** Robbin Gibb, Lara Coelho, Nicole Anna Van Rootselaar, Celeste Halliwell, Michelle MacKinnon, Isabelle Plomp, Claudia L. R. Gonzalez

**Affiliations:** ^1^Department of Neuroscience, University of Lethbridge, Lethbridge, AB, Canada; ^2^Holy Spirit Catholic School Division, Lethbridge, AB, Canada; ^3^Lethbridge School Division, Lethbridge, AB, Canada

**Keywords:** adult capability, assessment tools, cognitive function, motor function, socio-emotional competency, 3–5-year-old

## Abstract

In recent years, play has been shown to be a powerful means to enhance learning and brain development. It is also known that through play children enhance their executive function (EF) skills. Furthermore, well-developed EF in preschoolers has been shown to be an important predictor for later academic and life success. Armed with this information a program, Building Brains and Futures (BBF), for developing EF through play was designed for 3–5-year-old. The program consisted of 10 simple, fun, and interactive games selected to enhance various facets of EF. The 10 games included were: dimensional change card sort, lips and ears, block building, musical freeze, opposites, pretend play, red light/green light, shared project, Simon says, and wait for it. The program was implemented with a group of children shown to have challenges with respect to kindergarten readiness. The approach was first, to build adult capability by sharing knowledge of brain development, EF, and the importance of play with educators, caregivers, and parents. Second, to build skills in delivering the program in the school setting. Children engaged with the program of games for a minimum of 6 weeks. Their performance on a battery of direct measures of EF, language, and motor skills, were recorded before and after the program. The results showed improvement in all three domains. In addition, adopters of the BBF program reported it was easily and successfully integrated into their existing preschool curricula. The importance of intentional adult directed play in building developmental learning, including EF, is discussed.

## Introduction

Executive Function (EF) is a set of cognitive processes that help an individual regulate and adapt their behavior. The ability to focus, hold, and work with information in mind, filter distractions, and switch gears are part of this regulation and adaptation (Zelazo et al., 2016). Strong EF is like having an air traffic control system at a busy airport to manage the arrivals and departures of dozens of planes on multiple runways (Center on the Developing Child, Harvard University, 2021). EF allows an individual to focus on multiple streams of information at the same time, and revise plans as necessary. Acquiring the building blocks of these skills is one of the most important and challenging tasks of the early childhood years (Zelazo and Carlson, 2020). The opportunity to build further on rudimentary EF capacities is critical to healthy development through early, middle childhood, adolescence, and into adult life (Moffitt et al., 2011; Robson et al., 2020).

In an early description of the functional units of the brain, Luria (1980) described the prefrontal cortex, which supports the programming, controlling, and verifying activity, as the “brain’s executive.” Based on Luria’s description of the function of the prefrontal cortex, Shallice (1982) suggested that a multiple factor model of EF was necessary to explain high-level cognitive function. Lezak (1983), described four executive processes: goal formation, planning, carrying out goal directed plans, and effective performance which he coined “executive functioning.” Since these early descriptions, the number of research papers on EF has exponentially increased, but the notion of what constitutes EF, and whether it is composed of separate constructs (and which ones) or unitary, is debated.

Currently, it is widely accepted that there are three elements at the core of EF (Miyake et al., 2000): shifting, updating, and inhibition. In addition to these three core elements, some researchers propose that self-regulation, attentional control, planning, goal setting, and problem solving are more complex aspects of EF (Blair and Razza, 2007; Diamond and Lee, 2011). As Morra et al. (2018) point out, a problem with defining what EF is, is the lack of clarity within the terminology that defines it. They provide the following examples: “updating is confused with working memory, and shifting is confused with flexibility” (Morra et al., 2018). This illustrates the need to develop more precise terminology regarding EF: “Researchers should place a greater collective effort on terminological clarity” (Morra et al., 2018).

An individual is not born with EF abilities but rather, they develop over time, from birth to adulthood. There is no consensus yet as to which EFs are present early and which develop later. Similarly, there is no agreement as to whether the core elements of EF are fully dissociable. In an attempt to understand if EFs are dissociable, Miyake et al. (2000) investigated the strength of the relationship between shifting, updating, and inhibition in college-aged students. They found that “executive functions may be characterized as separable but related functions that share some underlying commonality,” Miyake et al. (2000) acknowledged that their findings may not apply to other populations including young children. In a systematic review, Karr et al. (2018) concluded that among preschoolers a unitary or bi-dimensional model best describe EF, whereas a nested factor model best described EF in adolescents and adults. Furthermore, Scionti and Marzocchi (2021) established that in preschoolers, a bi-dimensional model provided the best fit for their data. Based on these findings, we adopt the notion that EF in preschoolers is a bi-factorial construct with working memory and cognitive flexibility as one indistinguishable factor, and inhibition as the other (Monette et al., 2015; Scionti and Marzocchi, 2021).

Acquisition of EF skills in the early years of life are crucial for optimizing development. In fact, EF predicts kindergarten readiness; children with better developed EF perform better in the kindergarten setting (Willoughby et al., 2017). Moreover, children who perform better in kindergarten have better success throughout their academic career (Jones et al., 2015). In a longitudinal study, Alloway and Alloway (2010) tested children’s working memory and IQ in a battery of tests at two times: at 5 years of age and 6 years later. The second time the children were tested, numeracy and literacy assessments were included as measures of academic performance. The researchers found that working memory at 5 years of age, was a better predictor than IQ of academic performance 6 years later. EF is not only important for academic success as a multitude of studies have shown that good EF is associated with better physical and mental health, work productivity, and social competency (Riggs et al., 2006; Stichter et al., 2016). Jones et al. (2015) investigated kindergarten children’s pro-social skills or the intent to benefit others, and found they were significantly related to key outcomes for adolescents and young adults. Children with poor pro-social skills had more involvement with the criminal justice system, higher incidence of substance abuse, unemployment, and mental health problems. Another study showed that poor EF is associated with several antisocial behaviors, including emotional instability and physical aggression (McQuade et al., 2017). In fact, in a recent meta-analysis, Robson et al., 2020 looked at 150 studies and found that self-regulation at age 4 relates to a host of discrete outcomes. Better self-regulation is positively associated with school competency, engagement, and academic performance including math and literacy. Self-regulation was negatively associated with internalizing/externalizing problems, peer victimization, depressive symptoms, obesity, smoking, alcohol and substance abuse, and criminal behaviour. Given this knowledge, it is paramount to strengthen EF early in life.

A growing body of literature has demonstrated that EF can be enhanced throughout the lifespan, and in particular in pre-schoolers, with interventions and targeted training (Zelazo, 2020; for a review, see Scionti et al., 2020). For example, studies have demonstrated that interventions that feature cognitive exercises (e.g., Röthlisberger et al., 2012), street dancing (e.g., Shen et al., 2020), or musical training (e.g., Moreno et al., 2011) effectively enhance EF in preschoolers. In a study with children 3–6 years of age, the authors tested the efficacy of an play intervention using wooden blocks (Schmitt et al., 2018). This 7-week intervention with 14 sessions (i.e., twice a week) lasting 15–20 min each was done in a group setting with 2–3 children per group. Each group was given the set of wooden blocks and asked to build something according to the instructions given to them. Each week, the instructions became increasingly complex ranging from simple requests (e.g., “build a boat”) to more analytical demands that required the child to construct a 3D model from a 2D picture. Children were assessed in three tests of EF and three tests of mathematics. All children benefited from the program but those children whose parents had the lowest level of education showed the biggest improvements. Taken together, this evidence highlights that interventions for preschoolers can have powerful and positive effects on the development of EF.

One powerful way to strengthen EF is through play (for a review, see Yogman et al., 2018). According to Dr. Stuart Brown, “we are designed by nature to flourish through play” (Brown and Vaughn, 2009). The Oxford Lexicon (2021) ^1^ defines “play” as a verb that means engaging in activity for enjoyment and recreation rather than a serious or practical purpose. Play is known to develop and enhance independence, social interactions, cooperation, imagination, creativity, language skills, working memory, ability to follow instructions, problem solving skills, emotional control, and physical fitness (Yogman et al., 2018). Nearly all of these skills are components of EF, thus a play-based approach is an ideal means to develop EF. In 2015, Gibb et al. (2015) assembled a play-based program of games that were reported in the scientific literature to have value in enhancing EF. This program was adopted by a community-based group, Building Brains and Futures (BBF), and delivered in early childhood education programs to enhance EF in preschoolers. The BBF program used an evidence-based approach; the knowledge that intentional play is a driving force in building EF and establishing positive, engaged, relationships with adults.

The goal of the BBF program was to increase EF ability in preschoolers. In order to achieve this goal, the focus was to improve teacher/caregiver knowledge of these skills and have them actively participate with the children in the program of games. Training was provided for the educator/caregiver on use of the program before they implemented it in their classroom. The program included 10 different 5-min skill-building games. Each of these activities focuses on strengthening the two core domains of EF in preschoolers: working memory/cognitive flexibility and inhibition. The 10 games include: Dimensional change card sort, lips and ears, block building, musical freeze, opposites, pretend play, red light/green light, shared project, Simon says, and wait for it. A brief description of these activities can be found in the Materials and Methods section. For more information on each of these games, see Gibb et al. (2015); and the Building Brains Together webpage.^2^

For the evaluation component of the BBF program, we used parent/caregiver responses on a group of standardized surveys including the Ages and Stages Questionnaire (ASQ 3; Squires and Bricker, 2009) for typical child development (with motor, language, and cognitive assessments), the ASQ-SE (Squires et al., 2015) for social emotional development, and the Behavioral Rating Inventory of Executive Function in Preschoolers (BRIEF-P; Gioia et al., 2003). The results of these surveys demonstrated improvement in multiple domains and have been published elsewhere (Coelho et al., 2020). In the current study we moved away from assessing children’s development through parent responses, to incorporate direct measures of EF, language, and motor development. We used a comprehensive battery of tabletop measures that were developed in house (big and small blocks, and “grass or snow”) or by others, specifically the Peabody Picture Vocabulary Test-III (PPVT; Dunn and Dunn, 1997). The big and small blocks and the PPVT were included because our previous work has demonstrated strong relationships between motor, language, and EF development (Gonzalez et al., 2014a,b, 2018; Netelenbos et al., 2018; Coelho et al., 2020). In fact, we have proposed that sensorimotor and cognitive abilities are inextricably linked; cerebral lateralization for sensorimotor functions served as a foundation for the development of cognitive abilities and their hemispheric functional specialization (Gonzalez et al., 2018). Thus, the tests we used in the current study included assessment of motor, language, and executive functions. Furthermore, with regard to EF, our intention with the selected tests was not to isolate and assess any one component of EF, but rather to investigate if the program yielded any gains in overall EF.

## Materials and Methods

### Training for Early Childhood Educators/Caregivers

Ultimately, we recruited educators/caregivers and children from four sites (A, B, C, and D). We provided training for early childhood educators/caregivers for all participant sites through a 1-day workshop that focused on brain development, social connection, and EF in children. The purpose of the workshop was to build understanding in the adults working with children around these topics. In addition, explanations and practical demonstrations of the games and which EF each game promotes, were presented. Furthermore, we discussed variations of the games and how to integrate them into existing classroom routines with all participants. The investigators were available to answer questions and provide advice to the educators/caregivers throughout the school term as required. At the end of the post-testing period, site supervisors and educators/caregivers were invited to provide feedback about the program. The qualitative data from the feedback will be reported in a future paper.

### Participants

#### Pilot Control and Experimental Sites

Thirty preschool children were recruited from two different educational sites. Site A consisted of 14 participants (five males; mean age 42.2 months) that did not follow the BBF program (Control site). Site B consisted of 16 participants (10 males; 49.3 months) that followed the program for a minimum of 8 weeks (Experimental site). Encouraged by the preliminary results of the pilot sites which revealed the positive effect of the BBF program, we recruited a larger sample of children.

#### Expanded Experimental Sites

We originally recruited 68 participants from four educational sites (A, B, C, and D). Sites A and B were the same sites as in the pilot study, but because it was a new academic year, a different cohort of children were involved. There were 16 children from site A (seven males; 49.6 months), and 25 children from site B (13 males; 50.9 months), as well as 17 children from site C (six males; 57.4 months), and 10 children from site D (three males; 51 months). All four sites followed the BBF program. We report data on those participants that were available to us for both pre- and post-testing. In accordance with the declaration of Helsinki written consent was given by the parent/guardians prior to the start of the experiment.

### Procedure

#### Program

The BBF program was implemented at four early education sites. For preschoolers, each of the 10 different skill building games work on both domains of EF: working memory/cognitive flexibility and inhibition. They take around 5-min each to complete and were used on a regular basis by the educator/caregiver. To examine the effectiveness of the program, children were tested twice, once at the beginning of the school year (pre-test) and again at the end of the school year, with a minimum exposure for any participant of 6 weeks.

##### Dimensional Change Card Sort

The instructor shows the child a deck of cards that contain two dimensions: color and shape. The instructor informs the child that the deck can be sorted according to either. They then work with the child to sort the cards into both possible dimensions (color and shape), and eventually how to switch from one dimension to another.

##### Lips and Ears

Children receive either a picture of a pair of lips (indicates they are the speaker) or a picture of an ear (indicates they are the listeners). Only children with the lips are allowed to speak. Children take turns alternating lips and ears.

##### Block Building

The instructor places small (Lego®) or big (Duplo®) building blocks on a table surface in front of the child. Also, in front, there is a pre-built model. The child is asked to recreate a model from the pieces that are evenly distributed on the left and right sides of the table. The instructor should encourage the child to use their preferred hand to reach out for the desired blocks to build the model regardless of where the blocks are on the table.

##### Musical Freeze

For this game, the instructor choses a pose from a variety of poses depicted by stickmen. With music playing, the child is instructed to assume the pose that is revealed as soon as the music stops. A new pose is then chosen for the next musical interlude. Children must perform the pose correctly or else they will get counted out. The last person to perform the pose is also counted as out. The game can continue until there is a clear winner.

##### Opposites

After familiarizing a child with deck of picture cards and what they are depicting, the children are asked to respond with the “opposite” of what is depicted on the card. For example, if the child is shown a picture of “the sun” the child is expected to say “night” and when shown “the moon” the child should respond “day.” They should respond as quickly as possible.

##### Pretend Play

This game involves two children playing together and taking on a “role” (e.g., doctor and patient). They should be encouraged to play in an unstructured manner, but some guidance may be needed at the start. The children should switch roles halfway during the play bout.

##### Red Light and Green Light

This game involves the teacher/caregiver providing the children verbal or visual cues as to when they should move (green light) and when they should stop (red light). The children can also take turns being the ones to give the instructions. If the child fails to follow the cues, the child will be called out.

##### Shared Project

Children work in pairs to create either a picture or another form of constructive creation from assorted household materials (e.g., paper, boxes, and tape). They are encouraged to decide what and how is to be created thus, negotiating is required.

##### Simon Says

In this game, the adult stands in front of the children and instructs them to follow all actions that start with the words “Simon says.” For example, “Simon says put your hands on your head.” The child should then place their hands on their head. However, if the adult gives an action without saying “Simon says” the children must not complete that action. If the child does, then they are out.

##### Wait for It

The instructor dispenses a tasty treat to all the children. The children are instructed to refrain from eating it until the instructor says they are allowed to do so. If they wait until the instruction to eat the treat is given, they receive a second treat. If they cannot wait for the instruction they do not receive the second treat.

Some of these activities (lips and ears and shared project) have been adapted from the Tools of the Mind program (Bodrova and Leong, 2006). The “dimensional change card sort” was adapted from original protocol of Zelazo (2006), the “opposites” game is an adapted version of Stroop task of Gerstadt et al. (1994) and “wait for it” was adapted from famous marshmallow test of Mischel et al. (1989). In order to examine how effective the BBF program was, children were tested twice in a pre- and post-program design. The description of the games was previously published in Gibb et al., 2015 and Coelho et al., 2020.

### Pre- and Post-testing

Both pre- and post-tests were comprised of four different direct (table-top) assessments. These activities included big and small block construction, an opposite task (“grass or snow”), and the PPVT. The block construction tasks were designed to measure motor and EF, the “grass or snow” measures EF and the PPVT assess the extent of a child’s vocabulary. We recognize that these tabletop tasks do not measure single aspects of EF, and they place demands on other non-executive processes. In addition, three indirect (surveys) were given to the parents of the children that participated in the program; the results of these surveys have been previously reported (Coelho et al., 2020). The surveys included the Behavioral Rating Inventory of Executive Function in Preschoolers (BRIEF-P; Gioia et al., 2003), the Ages and Stages Questionnaire (ASQ 3; Squires and Bricker, 2009), and the ASQ-SE (Squires et al., 2015) for typical child development. Here, we report the GEC which refers to the global executive composite of the BRIEF-P and it measures overall EF. The ASQ is a standardized and commonly used measure of child development that covers five domains (communication, gross motor, fine motor, problem solving, and personal-social). The ASQ:SE measures socio-emotional development. These data were included to conduct correlation analyses between the direct and indirect measures.

### Big and Small Block Construction

**Figure 1:**
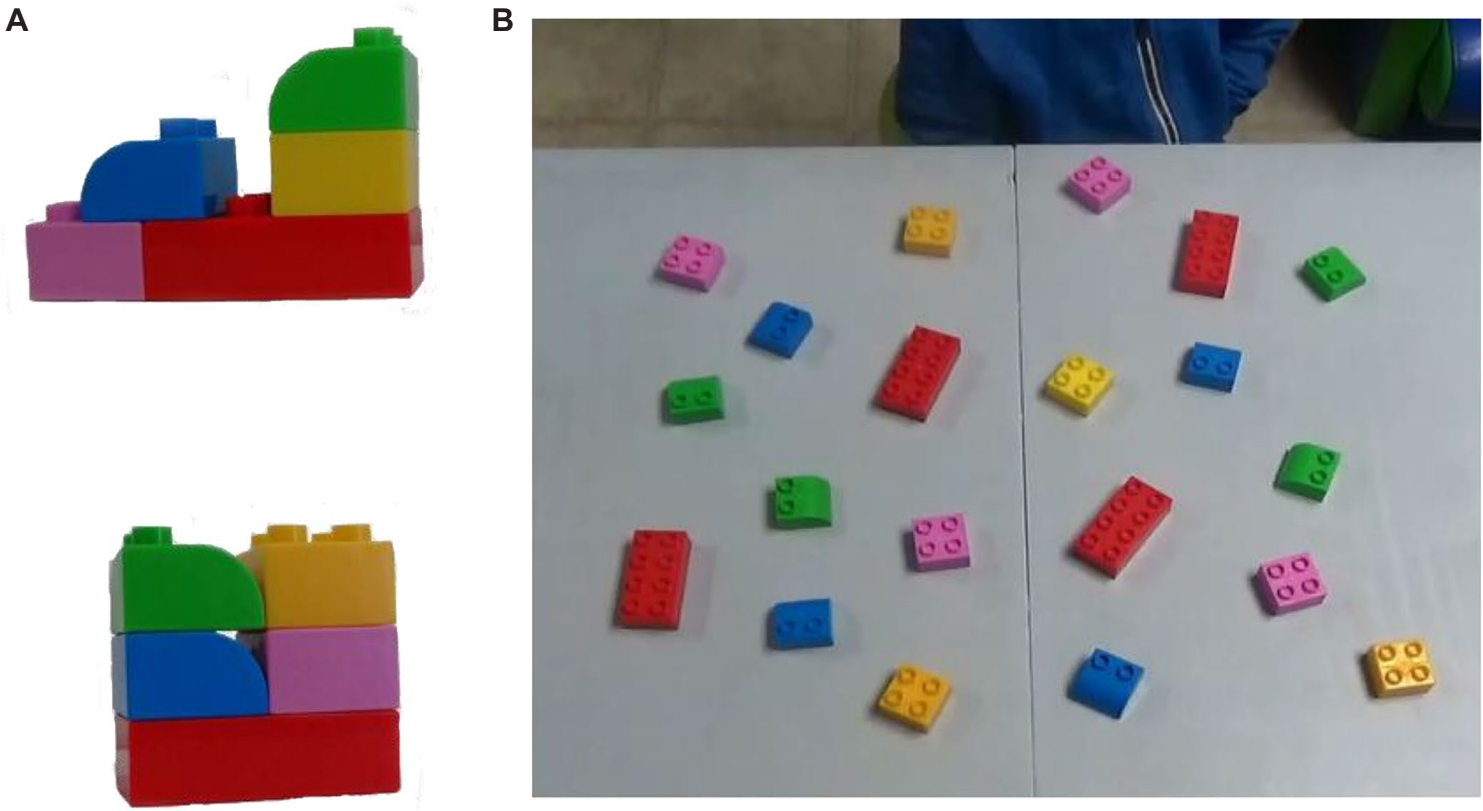
Frame **(A)** shows two examples of the big block models used in the study. Note how both models are constructed of the same five pieces. Frame **(B)** shows the set-up for the big block task. All five pieces for each model were available in each of the four quadrants.

**Figure 2:**
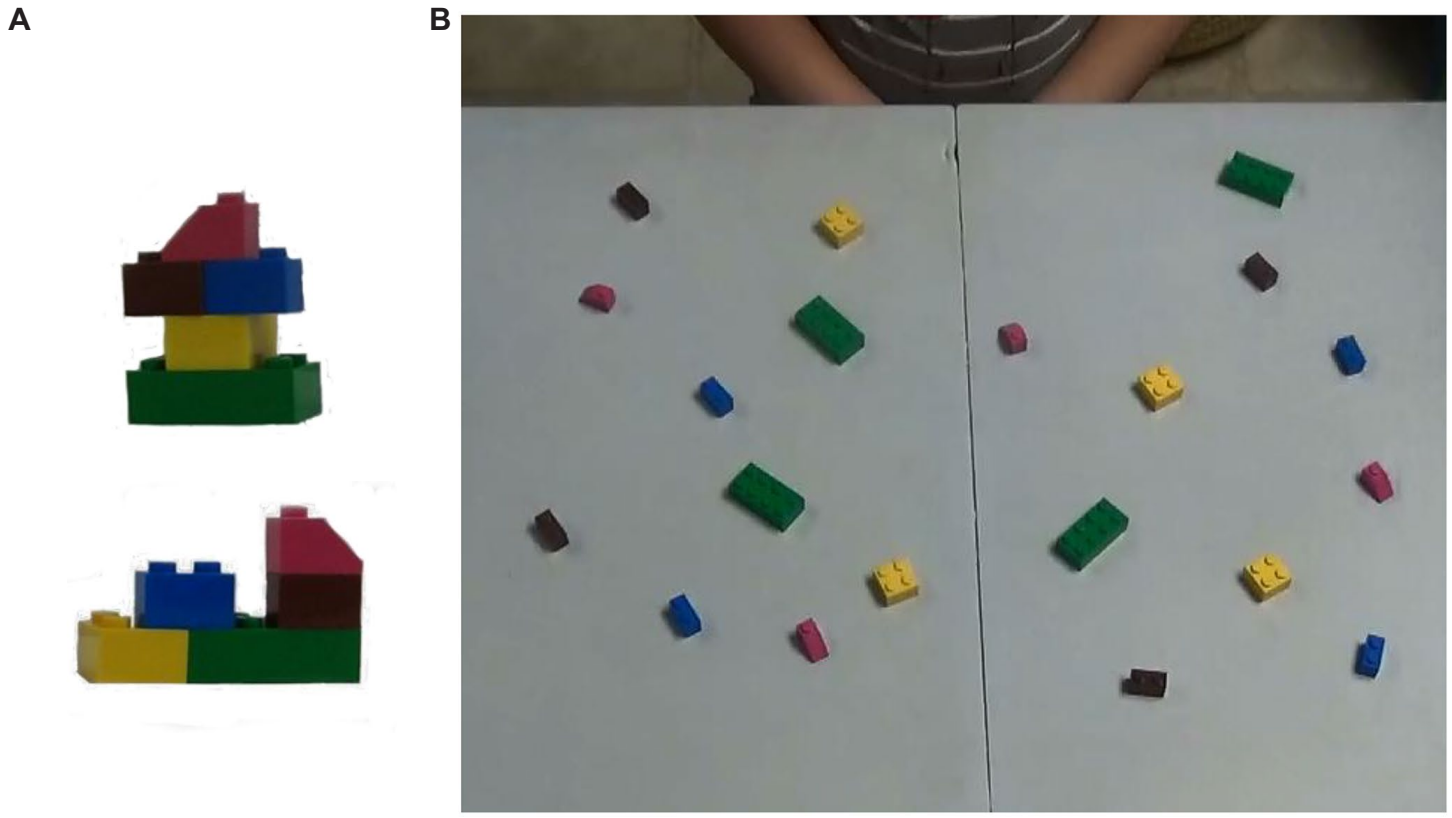
Frame **(A)** shows two examples of the small block models used in the study. Note how both models are constructed of the same five pieces. Frame **(B)** shows the set-up for the small block task. All five pieces for each model were available in each of the four quadrants.

Children completed two different grasp-to-construct tasks, one with big building blocks and one with small blocks. For both tasks, children were seated in front of a table, on a small stool. Twenty items (either large or small building blocks) were spread out on a 60 cm L × 80 cm W workspace (within arms-reach). This workspace was split into four unmarked quadrants with identical sets of five items located in each quadrant for even distribution of the blocks. The participants were unaware of this set up. For both the big block (see Figure 1) and small block (see Figure 2) tasks, participants were presented with four unique models for the child to replicate. Each model was comprised of the same five pieces; see Figure 1. The models were presented in succession directly across the table from the participant. The five pieces needed to complete each model were available in each of the four quadrants. We asked participants to replicate each model as accurately as possible. We did not provide children with any instruction on what speed to build or what hand to use. Once the participant had completed the first model, the second model was presented in order for replication. We did not put any additional pieces on the table, thus by the end of the fourth model there were no remaining pieces on the table. This procedure was identical to the one used in Gonzalez et al. (2014a,b).

#### Analysis

An important note to make regarding the grasp-to-construct tasks is that although we included them as a measure of motor development, the way the task is designed challenges EF in both domains. In order to successfully replicate the model presented, the child needs to make use working memory/cognitive flexibility to keep the instruction in mind, remember which blocks have been used and where to find the next block on the tabletop, and the ability to imagine what the model looks like from different perspectives. Although working memory/cognitive flexibility is the main EF for successful completion of the task, inhibition is also at play. The child must use inhibition to overlook blocks that “would do” but are not the exact one that is needed to make an identical copy of the presented model. In addition, the child must inhibit the desire to build their own model or to modify the presented model.

##### Latency

For the two grasping tasks (big and small blocks), we examined latency of the building time. For the grasp-to-construct tasks, this was calculated as the time each child spent building each of the four models added together for a total building time.

##### Errors

Mistakes were characterized as either placing the wrong piece on a Mega/Lego Block model, or by putting the piece in the wrong direction.

### “Grass or Snow”

**Figure 3:**
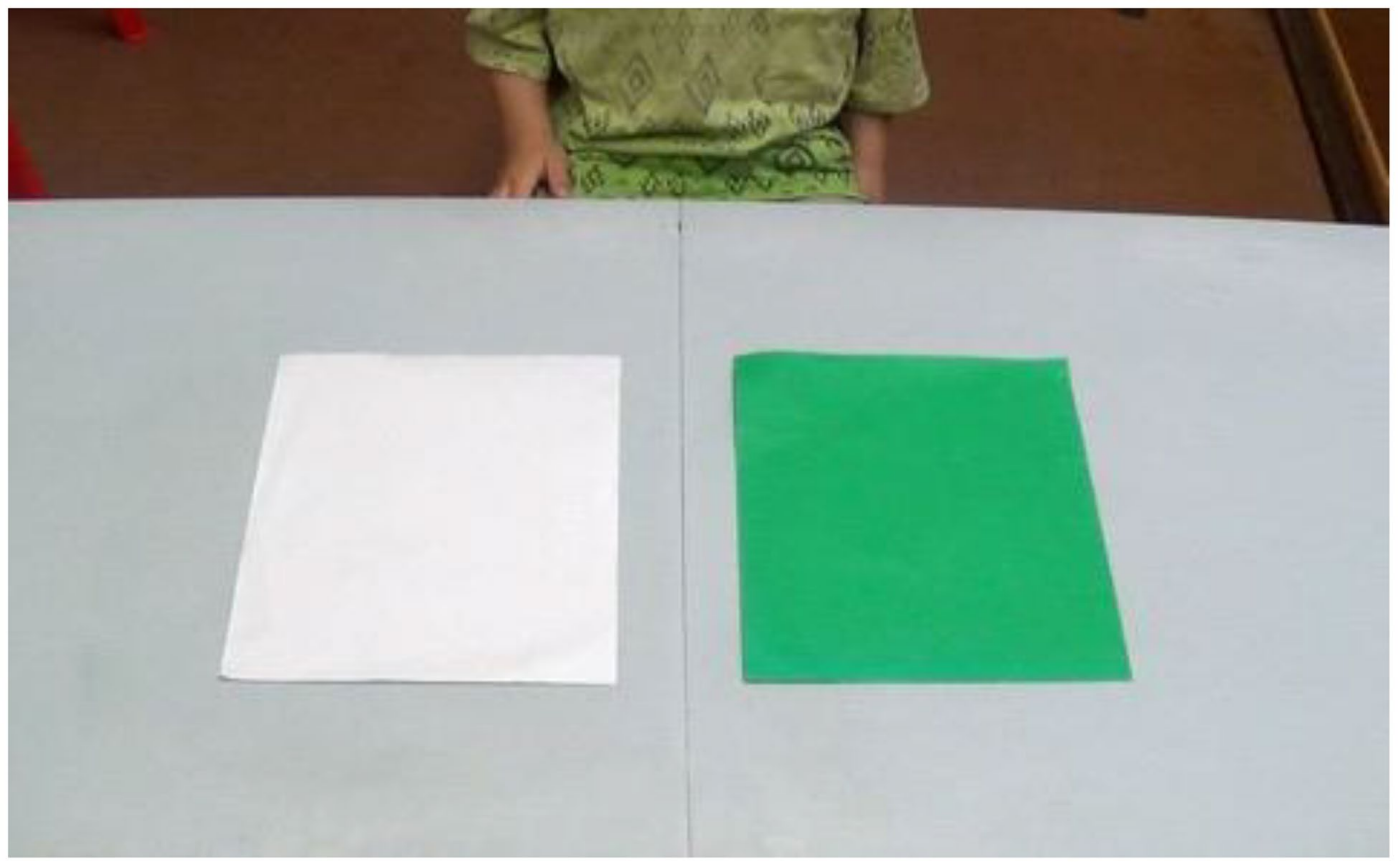
The grass or snow task. Children were required to point to the green square when the experimenter say “snow” and to the white square when the experimenter said “grass.”

Two laminated sheets of paper were presented to the child (see Figure 3). One of the sheets was green and the other was white. We explained to the child that for the purposes of this game, the white sheet represented the “grass” and the green sheet the “snow.” We then asked the child to point to either the grass or the snow from a pseudorandomized list of 10 commands (five to each target). Although this test requires working memory/cognitive flexibility as the child needs to hold in memory the instruction of what each sheet is meant to represent the emphasis is on inhibition. The child needs to embrace the notion that white represents grass, and that green represents snow and has to inhibit the urge to point to the color that matches the word (i.e., the green sheet when “grass” is called) and instead point to the incongruent sheet (i.e., point to the green sheet when “snow” is called).

#### Analysis

We expressed accuracy as percent correct and it was calculated as (total correct responses/total responses) × 100.

### Language Task

We used the PPVT (Dunn and Dunn, 1997) to discover the extent of each child’s vocabulary. The PPVT is a validated, widely used measure of children’s receptive vocabulary abilities.

**Figure 4:**
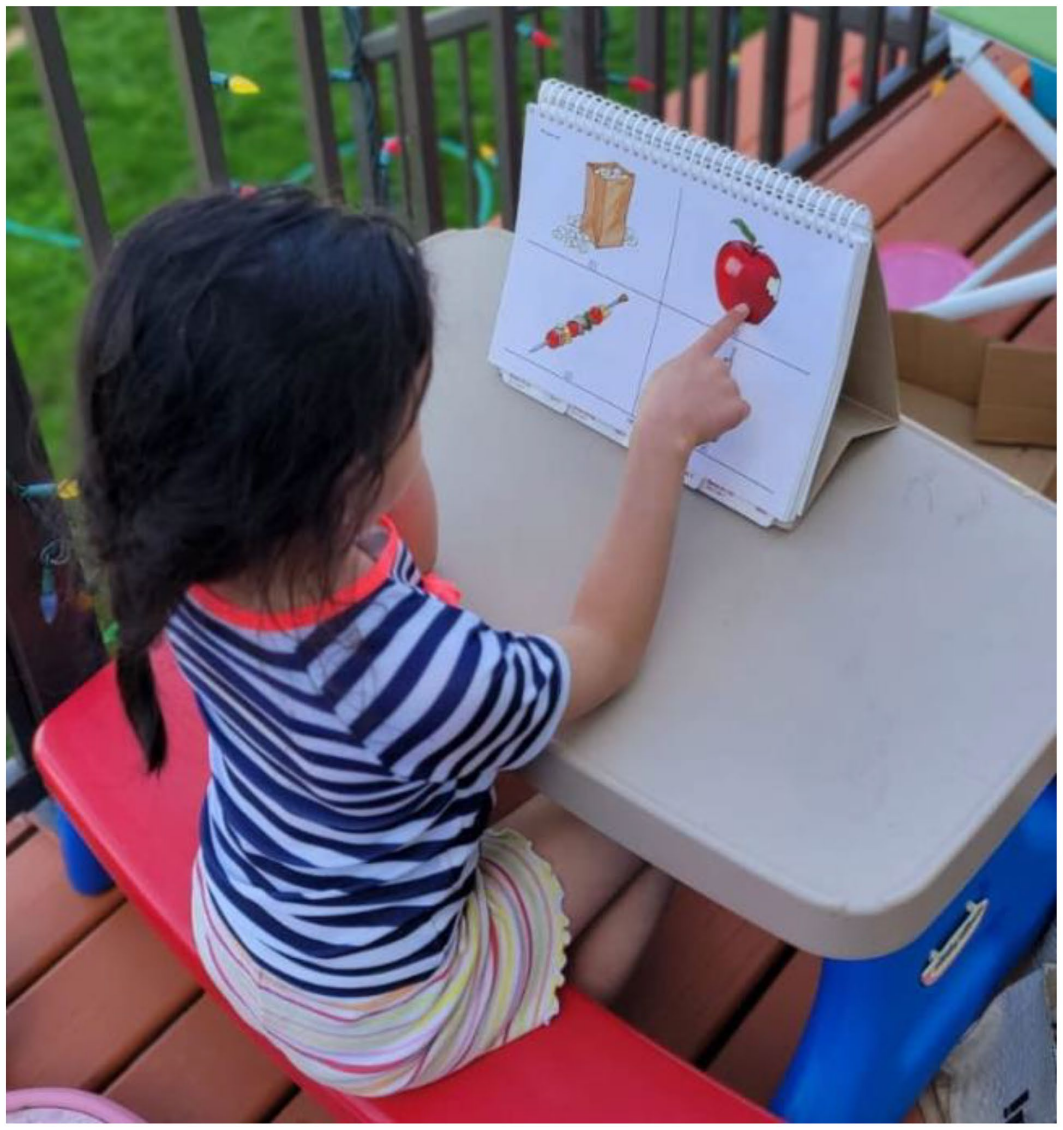
Peabody Picture Vocabulary Test-III (PPVT). The child hears the word “apple” and correctly points at the object depicting it.

To begin, the children sat down at a table with the PPVT centered in front of them (see Figure 4). Testing took approximately 10–25 min to complete. The PPVT was administered in accordance with the original protocol (Dunn and Dunn, 1997). Each child completed the age-appropriate practice page before starting the actual test. The practice page consisted of four different pictures. The experimenter read a word, and the child pointed to the picture that represented the stated word. Practice continued until the child correctly identified all four pictures on the practice page. The experimenter provided positive verbal reinforcement after each correct answer in the practice phase only. Similarly, during the test phase, the child saw a page containing four pictures, however, the experimenter only announced one word, and after the child pointed to a picture, the experimenter would continue to the next page. Each child began the test phase at the age-appropriate set of words and proceeded with the task until reaching the ceiling level (eight errors in one designated set of 12 words). If a child expressed uncertainty, and did not know the correct picture, the experimenter simply told them to “pick their best guess,” selecting the image they perceived as most likely correct. From the baseline set to the ceiling set/end of the test, the words consisted of multiple categories (nouns, verbs, and adjectives), and the level of difficulty increased with each set.

#### Analysis

The raw score on the PPVT was calculated as the number of correct answers from the total number of trials completed. This was then converted into the standard score by comparing the raw score values for the particular child’s age.

### Analyses

To determine if the program was of benefit to the children, a 2 × 2 pre/post testing repeated measures ANOVAs with the Control and Experimental sites as between factors were conducted. All statistical analysis were conducted with SPSS version 17.

We have included a table (Table 1) of the results of the indirect measures of development (the BRIEF-P, ASQ, and ASQ:SE) reported in Coelho et al., 2020. We conducted correlation analyses between these results and the outcomes of the direct measures (big and small block, the “grass or snow,” and the PPVT tasks). This was done to determine if parents perception of their child’s general development and EF was related to their child’s performance on the tabletop measures we used.

**Table 1:** The statistics for the questionnaires.

		BRIEF-P		ASQ						ASQ:SE	
		GEC		Comm.		Problem solving		Personal social		ASQ:SE	
		Mean	*SE*	Mean	*SE*	Mean	*SE*	Mean	*SE*	Mean	*SE*
Control group	Pre-test	52.9	3.0	48.2	3.5	53.2	3.4	48.9	3.1	41.8	6.3
	Post-test	53.5	3.5	55.7	1.7	54.3	1.4	51.1	2.6	34.2	7.1
	Value of *p*	0.82		<0.01		0.70		0.25		0.42	
BBF group	Pre-test	57.9	1.8	47.1	1.7	48.9	1.6	49.3	1.6	52.1	5.3
	Post-test	55.3	1.8	51.8	1.4	51.8	1.6	54.1	1.3	42.9	5
	Value of *p*	0.02		<0.01		0.05		<0.01		0.03	

*These results have been published elsewhere (Coelho et al., 2020). The children in the BBF program improved on the GEC portion of the Behavioral Rating Inventory of Executive Function in Preschoolers (BRIEF-P), and additionally showed improvements on the communication, problem solving and personal social components of the Ages and Stages Questionnaire (ASQ), and the ASQ:SE (lower values in the ASQ: SE indicates better performance)*.

## Results

### Pilot Control and Experimental Sites

The 2 × 2 repeated measures ANOVAs with pre/post testing as within factors and group (Control versus Experimental) as between factors showed:

#### Big Block

For errors, here was a main effect of session [*F* (1,19) = 14.6, *p* = 0.001]. Participants made fewer errors in the post-testing (1.5 ± 0.4) compared to the pre-testing session (6 ± 1.3). No other main effect or interactions were found. No main effect or interactions were found for latency.

#### Small Block

For errors, there was a main effect of session [*F* (1,19) = 10.6, *p* = 0.004]. Children made on average 6.8 ± 1.2 mistakes in the pre-test. This was reduced to 2.9 ± 0.78 at the post-test. No other main effect or interactions were found. No main effect or interactions were found for latency.

#### “Grass or Snow”

There were no significant results.

#### PPVT

There was a main effect of session [*F* (1,20) = 4.5, *p* = 0.05] and a Session × Site interactions [*F*(1,20) = 6.5, *p* = 0.02]. There was no significant change in score for the control group (pre-test: 114.1 ± 3.3; post-test: 113.4 ± 3.5). For the experimental group, there was an improvement from the pre-test (101.5 ± 4.4) to the post-test (109.9 ± 4.7).

### Paired-Samples *t*-Tests

We based our decision to not complete an ANOVA’s between the control and experimental groups because of the differences in sample sizes between the two groups (*n* = 14, *n* = 61, respectively). Unequal sample sizes can affect the robustness of the equal variance assumption (Keppel, 1993), and the statistical power of any ANOVA performed will be greatly reduced. In addition, any effect that we find in the ANOVA may be driven by the larger sample size in the experimental group. For instance, if we find that children in the experimental group outperform those in the control, it may just be reflecting the fact we had more 4-year-old in this group. So this would be reflecting a main effect of age, and not a main effect of group. Thus, we conducted separate paired samples *t*-tests to further examine possible changes in performance on table-top assessments of EF due to exposure to the program.

#### Control Site

There was a significant difference between the pre- and post- testing (after Bonferroni corrections) on the number of errors in the grasp-to-construct with small blocks task [*t*(13) = 3.5, *p* = 0.032]; children made significantly fewer errors at post-assessment. However, there were no improvements in the time to complete the big blocks (*p* = 0.2) or small blocks (*p* = 0.4) tasks. As for errors, there were no improvements in the grasp-to-construct with big blocks task (*p* = 0.1), and the “grass or snow” (*p* = 0.47). There was no improvement on the PPVT (*p* = 0.74) either.

#### Experimental Site

*T*-tests (after Bonferroni corrections) revealed that children made fewer errors in the big block task [*t*(15) = 3.1, *p* < 0.01], the small block task [(*t*(15) = 2.6, *p* = 0.02)], and the “grass or snow” task [grass snow; *t*(10) = −2.6, *p* = 0.03]. Results of the PPVT were not significant but a trend was noted [*t*(14) = −1.6, *p* = 0.14] in that children scored better at post-testing.

As the shown in Table 2, performance in the EF tasks (“grass or snow”) was higher in the Control site at pre-test. No improvement was observed at post-test in the Control site, but significant improvement was seen in the Experimental site.

**Table 2:** Means and SEs for all tasks in the Pilot Control and Experimental sites and for the Expanded Experimental sites.

	Pilot control				Pilot experimental				Expanded experimental			
	Pre-test		Post-test		Pre-test		Post-test		Pre-test		Post-test	
	Mean	*SE*	Mean	*SE*	Mean	*SE*	Mean	*SE*	Mean	*SE*	Mean	*SE*
Small Block Errors	8.6	1.2	3.6	1.0	5.0	2.2	2.3	0.8	5.1	0.6	3.0	0.5
Small Block Time	257.4	27.7	241.6	23.5	211.1	38.1	215.9	40.1	168.7	9.7	167.1	11.2
Big Block Errors	5.2	1.3	1.3	0.5	6.7	2.4	1.7	0.7	4.8	0.9	1.6	0.3
Big Block Time	152.3	22.6	138.6	28.4	172.8	25.2	141.4	36.8	160.4	11.0	105.8	7.9
“Grass or Snow” (% correct)	42.7	12.5	58.6	17.4	26.0	10.3	68.3	16.4	52.1	4.4	74.7	4.1
PPVT (standard score)	114.1	3.4	113.4	3.1	101.5	4.1	109.9	5.5	100.5	2.0	102.0	1.9

### Expanded Experimental Sites

We only conducted paired samples *t*-tests (pre/post testing) on the Expanded Experimental (*n* = 68) because an analysis of this group against the 12 control participants from the pilot would be inappropriate.

**Figure 5:**
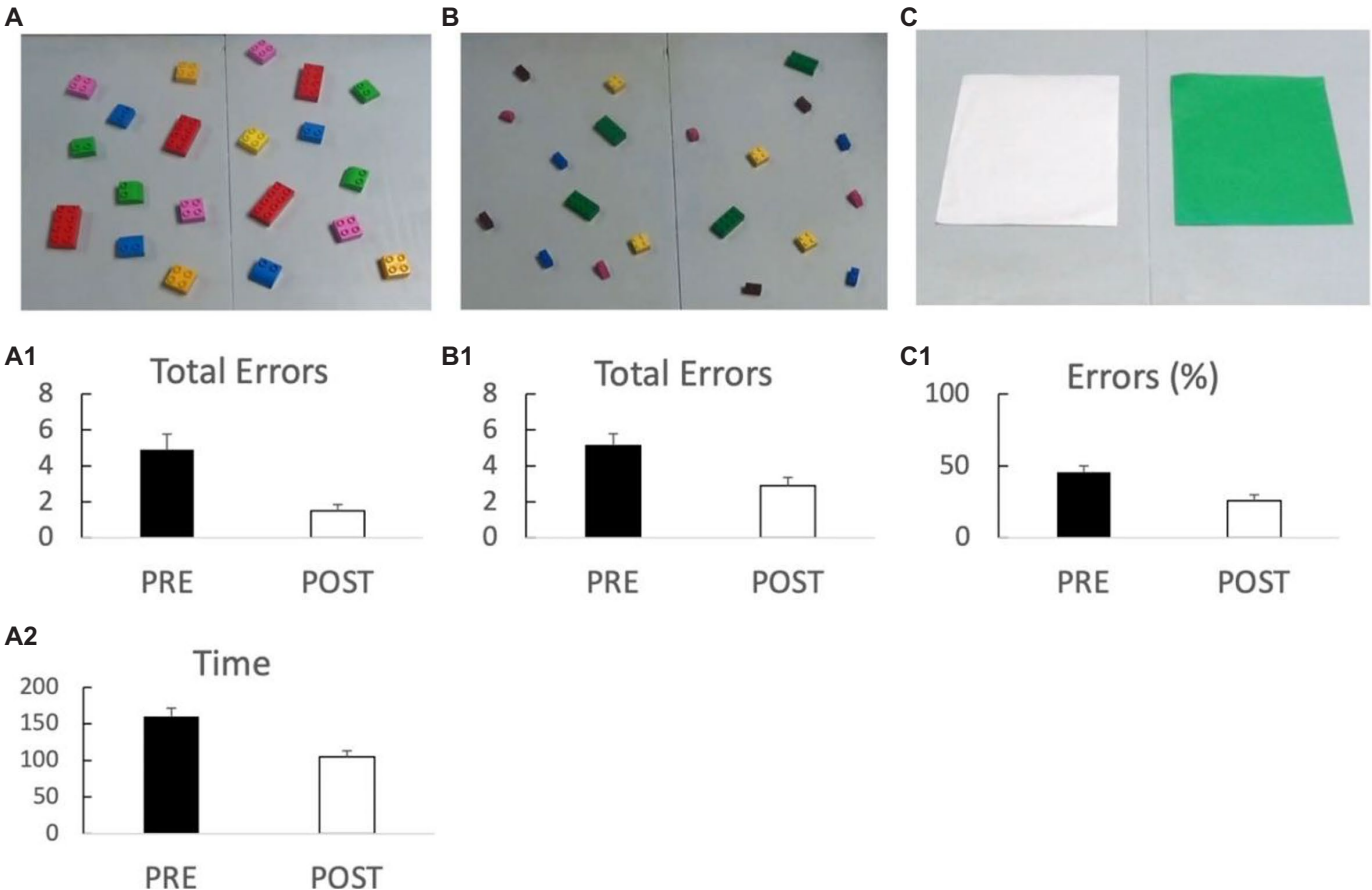
Significant results from the Building Brains and Futures (BBF) Pre-Post-testing. Panel **A** shows the results of the big block task with **(A.1)** showing errors and **(A.2)** showing time to complete the task. Panel **B** illustrates the results of the small block task and **(B.1)** the errors. Panel **C** shows the results of the “grass or snow” task and **(C.1)**. the percent incorrect. In all cases, *p* < 0.001 Bonferroni corrected.

After Bonferroni corrections, paired samples *t*-test (pre/post testing) on all table-top measures revealed that post-program the children in the Expanded Experimental sites showed significant improvements in the following measures (see Figure 5): big block errors [*t*(55) = 4.3, *p* < 0.001], small block errors [*t*(53) = 2.2, *p* < 0.001], big block time [*t*(48) = 5.9, *p* < 0.001], and “grass or snow” errors [*t*(49) = −3.6, *p* < 0.001]. No significant difference was found for the PPVT (*p* = 0.26).

### Correlation Analyses Between Direct (Tabletop) and Indirect (Surveys) Measures

As shown in Tables 3A,B, there were significant correlations between the direct (tabletop, current manuscript) measures of EF and those reported by parents on the BRIEF-P, ASQ, and ASQ-SE (published data, Coelho et al., 2020). The results from the correlation showed that in all cases the better the performance on the tabletop measures, the better the reported behaviour on the questionnaires. For example, the time to complete the big block task, significantly correlated with the communication, gross motor, problem solving, and personal/social subscales of the ASQ; the faster the child assembled the block models, the better the parent’s ratings for that child on the ASQ (Coelho et al., 2020).

**Table 3:** Table 3A shows the correlation between the pre-test scores on the tabletop tasks and the questionnaires and Table 3B shows the correlation between the post-test scores on the tabletop tasks and the questionnaires (BRIEF-P, ASQ, ASQ:SE).

Table 3A							
	BRIEF-P	ASQ					ASQ:SE
	GEC	Communication	Gross motor	Fine motor	Problem solving	Personal Social	ASQ:SE
Small block errors	0.2	0.21	0.15	−0.02	−0.01	0.01	0.15
Small block time	0.00	−0.17	**0.29** ^*^	**−0.31** ^*^	−0.13	0.04	**0.29** ^*^
Big block errors	0.13	−2	0.14	−0.05	−0.14	−0.18	0.14
Big block time	−0.08	−0.15	0.18	−0.20	−0.15	−0.20	0.18
Grass or Snow	0.15	**−0.35** ^**^	0.12	−0.16	−0.17	−0.19	0.12
PPVT	−0.17	**0.41** ^**^	−0.8	**0.40** ^**^	**0.53** ^**^	**0.26** ^**‡**^	−0.8
Table 3B							
Small block errors	0.10	−0.2	−0.02	−0.09	−0.04	0.06	0.05
Small block time	0.10	−0.02	**0.34** ^*^	−0.15	−0.17	−0.07	0.09
Big block errors	0.21	−0.23	−0.15	−16	0.01	0.12	−0.8
Big block time	0.15	**−0.39** ^**^	**−0.44** ^**^	−0.24	**−0.30** ^*^	**−0.29** ^**‡**^	0.01
Grass or Snow	0.2	**−0.37** ^**^	**−0.42** ^**^	**−0.32** ^*^	−0.23	**−0.52** ^**^	**−0.32** ^*^
PPVT	**−0.45** ^**^	**0.6** ^**^	0.21	**0.31** ^*^	**0.59** ^**^	**0.43** ^**^	**0.49** ^**^

Bolded values are significant at these levels;

* *p < 0.05*.

** *p < 0.01*.

‡ *p < 0.1*.

## Discussion

Executive Function is a set of cognitive processes that help an individual regulate and adapt their behavior. Well-developed EF has been identified as a key component of academic and life success. BBF was a community program with a focus on strengthening EF in preschoolers. The program included 10 different short skill-building games. Each of these activities focus on strengthening EF. The 10 games include: red light green light, Simon says, opposites, musical freeze, pretend play, lips and ears, shared project, wait for it, dimensional change card sort, and right is right.

The BBF program had two main goals: First, to engage and share knowledge about brain development and EF with educators/caregivers, and to familiarize them with the BBF games and their importance in building EF. Second, to implement the games at four test sites. In the current study, we assessed children’s EF with tabletop measures before and after engagement with the games. The results showed improvement in EF, but also in motor and language (albeit modest) aspects of development. These findings nicely align with our previous study (Coelho et al., 2020) using surveys of EF and child development. In Coelho et al. (2020) the development of pre-school children before and after the BBF program was assessed through parent reports on three standardized questionnaires (BRIEF-P, ASQ, and the ASQ-SE,) designed to measure child development in motor, cognitive, and social domains. Significant improvements in various measures within all three questionnaires were seen in children in the BBF program only.

A corollary of the current study was the unique opportunity to directly compare the results of the tabletop measures and those of our previously published parent surveys (Coelho et al., 2020). Although our research has used the big and small block tasks extensively (Stone et al., 2013; Gonzalez et al., 2014b, 2015), these tasks along with the “grass or snow” had not been validated as measures of child development and EF in particular. The results of these correlation analyses demonstrated that after the intervention, children’s tabletop measures of EF including the big and small block task, correlated with parent surveys (see Tables 3A,B). For example, “grass or snow” correlated with the communication, gross motor, fine motor, and personal/social subscales of the ASQ and with ASQ-SE. In all cases, the fewer the errors made in the “grass or snow,” the better the performance as reported on both ASQ surveys. For the block tasks, we saw correlations with the ASQ on communication, gross motor, problem solving, and personal/social subscales. Here, the faster the child was at assembling the block models, the better their performance on the ASQ. Finally, the PPVT significantly correlated with the BRIEF-P global executive composite and with the communication, fine motor, problem solving, personal/social on the ASQ and with the ASQ-SE; better performance on the PPVT was related to better performance on the questionnaires. As Tables 3A,B show, some of these significant correlations were present before beginning the BBF program, but following the program many more were identified and they were stronger. Together, these results suggest that the BBF program given to preschoolers may broaden the transfer in near (i.e., EF) and far (non-EF domains) domains. A recent meta-analysis (Scionti et al., 2020) demonstrated that interventions that target EF have more widespread transfer than previously thought.

The first finding in the current investigation is the difference in the pre- and post-testing between the Control and Experimental sites in the pilot study. Educators/caregivers at both sites, A and B, were trained at the workshop described in the methods, but site A (Control) did not follow the BBF program whereas site B (Experimental) did. The only significant improvement in children’s performance between pre- and post-testing at site A, was on the number of errors on the small block task. In contrast, there were significant gains in a number of assessments for the children at site B: Children made fewer errors on both the big and small block tasks, and in the “grass or snow” task. We also saw a trend for improvement in the PPVT. Encouraged by these results, we expanded the program in the next academic year to include two additional sites and a new group of children in sites A and B.

When all four sites participated in the program (Expanded Experimental), we found strong evidence of improvement in exactly the same measurements that showed improvement in the experimental site of the pilot; fewer errors in the big and the small block tasks, and in the “grass or snow” task. This finding nicely aligns with our previous report on the effect of the program on standardized surveys of EF, language, motor, and social development (Coelho et al., 2020). Although the difference between the pre- and post-assessment did not reach significance for the PPVT, the trend was in the expected direction. It is important to note that one of our sites was a First Nations pre-school, and it is not clear if the PPVT in its standard form is appropriate to assess vocabulary in this population (Eriks-Brophy, 2014). It is often the case that First Nations children speak English as their second language, and this may have contributed to the lack of significant effects after the program. As yet, the appropriate tools (e.g., a translation of the PPVT into Blackfoot) are not available to address this point.

The 10 games that comprise the BBF program focus on working memory/cognitive flexibility and inhibition which are the two core domains of EF in preschoolers. When creating the BBF program, we carefully chose the games to maximize effects on EF by using a multifaceted approach. Some of the games demand more physical effort, some more social interaction, and others more problem solving. However, all of them feature an emphasis on building working memory/cognitive flexibility, and inhibition. As mentioned in the introduction, there is a growing body of literature that demonstrate intimate relationships between motor and language function (Gonzalez et al., 2014a; Forrester and Rodriguez, 2015; van Rootselaar et al., 2020, 2021), motor and EF skills (Gonzalez et al., 2014a; Luz et al., 2015), and EF and language (Kuhn et al., 2014; Miller and Marcovitch, 2015; Netelenbos et al., 2018). In fact, Tables 3A,B illustrate this point. The outcomes of measures for the three domains, were significantly correlated.

An important aspect of the BBF program is featuring a winner/loser dynamic in which only one child can win the game. This challenges inhibition as self-regulation is required to accommodate the concept of loss. Three of the games in the BBF curriculum (musical freeze, red light, green light, and Simon says), emphasize this point. It is important to introduce children to losing at an early age, so they can accept losses throughout life. Furthermore, when a child is experiencing emotional dysregulation after a loss, other children in the group often exhibit prosocial skills to support their peer and help them feel better (Perner and Lang, 1999; Carlson and Moses, 2001; Jones et al., 2015). One of our educators provided an example on this point: *“Cheering for the winner: Not everyone can win every time. This is an important concept for children to learn, sometimes they will not win but we can still be happy for who has won. This is hard for some children, but most have enjoyed the cheering. As time [goes] on they are becoming more and more accepting of not winning every time.”*

One of the key aspects of the BBF program is the use of adult-directed intentional play as a tool to strengthen EF skills and build meaningful relationships. Traditional views of play argue that self-directed play builds developmental learning including EF, whereas adult-directed play builds academic learning (Salisbury et al., 2017; Sim and Xu, 2017). The results from the current study suggest another possibility: Adult-directed intentional play builds developmental learning such as EF and by enhancing EF, the skills for later academic learning are strengthened (Jones et al., 2015). Within the BBF program, the adult may start as a leader, but roles can be reversed, and the child may take turns as a leader further developing executive control. Furthermore, in the BBF program, the adult is an active participant in the game. In doing this, the adult is building relationships and attachment with the children by prioritizing playing with them. We believe that this is a side benefit of the BBF program; the improvement in the quality of the child-adult relationship. By spending playful time engaged with an adult, children develop stronger positive relationships with these adults which in turn can strengthen EF (Yogman et al., 2018). In future studies, we will investigate the influence of game playing on the quality of the adult/child relationship and its effect on children’s EF.

Two important advantages of the BBF program are its ease of integration into pre-existing early education programs and that all children can participate at the same time. All classrooms at the four BBF test sites embraced the program and quickly incorporated the games into their daily routines. Importantly, not all games were played every day. The only instruction given to educators/caregivers during the workshop was to play at least one game a day but to rotate through all of them on a regular basis. In other words, there was tremendous flexibility for the educator/caregiver to incorporate the BBF program into their learning environment as they saw fit. It is significant to note, that two of the educators involved with the BBF program have won prestigious awards at the provincial and national level as a result of their work with the BBF. As it turns out, adherence to the program was easy for them. The other strength of the BBF program is that it is administered in a group setting; in the classroom with all children participating. A recent systematic review and meta-analysis, concluded that interventions to improve EF are more effective when conducted in groups (Scionti et al., 2020).

From the feedback requested at the end of the program we include two comments; the first one from the supervisor at one of our sites and the second from a grandparent of one of the participants:

*“Play-based activities from the Building Brains program promote engagement, fun, and positive growth in executive functioning skills. These activities can be implemented throughout the programming day in easy and effective ways. Parents are invited to participate in the classroom to learn the activities which can be easily done in the home environment. The research behind the use of the Building Brains program demonstrates that children can make effective gains in executive functioning skills within a few months of consistently using the activities.” Isabelle Plomp. Early childhood coordinator, Lethbridge School Division*.

*“I certainly hope this program continues, not only continues but expands to be in every school throughout Alberta, throughout Canada. If we can start this at a young age like they are here we are going to see huge, huge differences in our children and their learning abilities, their abilities to function out in society.” Comment from a grandparent of one of the child participants*.

### Limitations

We recognize that one of the biggest limitations of the current study is that when we expanded to include four experimental sites, we did not include a control site. The preliminary results were received with great interest and enthusiasm by the local school districts and as such they all wanted to take part in the program. We embraced this unique opportunity. We have continued the expansion of the program, and this includes a large control site.

Another limitation, as is the case in the vast majority of field work, is the reporting of average data. We recognize that whereas some individuals showed significant improvement in one or more of the behavioral measures, others did not. We continue to examine the data to better understand the moderators that affect individual child outcomes including developmental delays, SES, parent education, and adverse prenatal and childhood experiences. We hope to provide these data in a future report.

Dosage is another limitation of the current study. Although all the educators that participated in the program committed to use at least one game per day (“minimum dose”) covering all 10 games throughout the term, we know that some played several of the games each day. This makes it difficult to determine exactly what would be the “optimal” dosage. We were hesitant to establish an upper limit for the number of games played each day, but rather wished to leave it to the discretion of each educator so the program remained flexible and easy to integrate into their established routines.

Assessment of EF is difficult because a concise description of it and how it develops is still under debate. We acknowledge that our testing measures were limited and not “pure” in that they assess more than one aspect of EF and they place demands on other non-executive processes. Moving forward, we will include more targeted measures such as the backward digit span task to measure working memory/cognitive flexibility and go-no-go task for inhibition.

Despite these limitations, the BBF program is free, easy to use, and as shown here, effective in building EF skills. As we continue to expand this program including a larger control and more experimental sites, we hope to gain a better understanding of factors (e.g., SES, gender/sex, adverse childhood experiences, etc.) that moderate child development so the BBF can include individualized approaches.

## Data Availability Statement

The raw data supporting the conclusions of this article will be made available by the authors, without undue reservation.

## Ethics Statement

The studies involving human participants were reviewed and approved by University of Lethbridge Human Subject Research Committee. Written informed consent to participate in this study was provided by the participants’ legal guardian/next of kin.

## Author Contributions

RG and CG conceived the project and wrote the paper. LC, NR, and CH did data collection and analyses. MM and IP facilitated access to the classrooms, ensured fidelity of program delivery, and provided information to educators and parents regarding the program. All authors contributed to the article and approved the submitted version.

## Funding

This work was supported by Palix Foundation, Anonymous Donor, City of Lethbridge FCSS, University of Lethbridge strategic operating fund, and National Science and Engineering Council of Canada (NSERC).

## Conflict of Interest

The authors declare that the research was conducted in the absence of any commercial or financial relationships that could be construed as a potential conflict of interest.

## Publisher’s Note

All claims expressed in this article are solely those of the authors and do not necessarily represent those of their affiliated organizations, or those of the publisher, the editors and the reviewers. Any product that may be evaluated in this article, or claim that may be made by its manufacturer, is not guaranteed or endorsed by the publisher.

## Footnotes

^1^ https://www.lexico.com/definition/play

^2^ www.buildingbrains.ca
